# Expression of the cannabinoid type I receptor and prognosis following surgery in colorectal cancer

**DOI:** 10.3892/ol.2012.1081

**Published:** 2012-12-18

**Authors:** CHAN KWON JUNG, WON KYUNG KANG, JAE MYUNG PARK, HYO JUN AHN, SANG WOO KIM, SEONG TAEK OH, KYU YONG CHOI

**Affiliations:** 1 Departments of Hospital Pathology, College of Medicine, The Catholic University of Korea, Seoul 137-070, Republic of Korea; 2 Surgery, College of Medicine, The Catholic University of Korea, Seoul 137-070, Republic of Korea; 3 Internal Medicine, College of Medicine, The Catholic University of Korea, Seoul 137-070, Republic of Korea

**Keywords:** cannabinoid receptor, survival, Kaplan-Meier analysis, stage, metastasis

## Abstract

The cannabinoid system has been considered to be a potential target of colorectal carcinoma therapy. The aim of this study was to address the correlation between cannabinoid type 1 (CB1) receptor expression and disease severity/outcomes in patients with colorectal cancer (CRC). CB1 receptor expression was analyzed by immunohistochemistry using tissue microarrays in consecutive patients who underwent surgical resection (n=534). CB1 receptor expression was categorized as a high (≥66%) vs. low (<66%) immunopercentage as a median split, and was analyzed in relation to disease severity and overall survival. CB1 receptor expression was observed in 409 patients (76.6%). Low CB1 receptor expression was more frequently identified in stage IV than in stage I/II or III cancer (P<0.01 for both). In stage IV CRC, high vs. low CB1 expression was correlated with a statistically significant poorer overall survival (P=0.033) that was independent of age, R0 resection, tumor differentiation and chemotherapy [hazard ratio (HR), 1.805; 95% confidence interval (CI), 1.042–3.094; P=0.035]. However, CB1 expression was not observed to be correlated with patient survival following surgery in stage I/II or III cancer. The high immunoreactivity of the cannabinoid type 1 receptor is a significant prognostic factor following surgery in stage IV CRC.

## Introduction

Cannabinoids have been implicated in physiological and pathological conditions including inflammation, immunity, analgesia, neoplasia and others (1). These associations have increased the interest in cannabinoids in recent years. The effect of cannabinoids in colorectal cancer (CRC) has been demonstrated in *in vitro* experiments and animal models, which indicate the antiproliferative, apoptotic and antimetastatic actions of cannabinoid agonists (2–5). In accordance with these observations, the levels of the major endogenous cannabinoids have been identified to be 2- to 3-fold higher in CRC than in the neighboring normal mucosa (5).

Antineoplastic effects are mediated by the activation of cannabinoid type I (CB1), type 2 (CB2) or a non-cannabinoid receptor-mediated mechanism (6). Among the receptors, the mechanism of tumor cell apoptosis has been rigorously investi gated by several research groups who have studied the CB1 receptor (2–5). CB1 is abundantly expressed in the brain and in numerous peripheral neurons (7). It is also found not only in normal colonic epithelium, smooth muscle and the submucosal myenteric plexus (8), but also in several colon cancer cell lines (6). Expression levels of CB1 receptors are downregulated in cancer compared with adjacent normal mucosa (3). Loss or inhibition of the CB1 receptor has been demonstrated to accelerate intestinal adenoma growth, whereas activation of the CB1 receptor attenuated intestinal tumor growth by inducing cell death via downregulation of the anti-apoptotic factor, survivin, in a genetic model of CRC progression (3). CRC patients who are either homo- or heterozygous for the 1359 G/A CB1 receptor polymorphism exhibit a shorter survival time compared with G/G wild-type patients, although the post-transcriptional mechanism has not yet been delineated (9).

The evidence mentioned thus far suggests that the endogenous cannabinoid system is able to regulate cancer progression and affect disease progression and outcomes. Although there have been a small number of studies concerning the prognostic role of the CB1 receptor in human tissues (10–12), the results were discrepant and CB1 expression of CRC has only been addressed in a single study (13). We hypothesize that increased CB1 receptor expression may be associated with decreased disease severity and more favorable clinical outcomes. Therefore, the aim of the present study was to investigate the correlation between disease severity/clinical outcomes and the expression level of the CB1 receptor.

## Materials and methods

### Patients

Between January, 2004 and December, 2007, a total of 544 consecutive patients with CRC who underwent surgery at Seoul St. Mary’s Hospital, Seoul, Korea, were enrolled. Patients who had surgery-related mortality (n=10) were excluded from this study; therefore, the clinical data of 534 patients were analyzed. The patients were followed up after surgery at regular 3- to 6-month intervals during the first year and then at 6-month intervals thereafter. Abdominal computed tomography was performed annually for the first 3 years and colonoscopy was performed every 1–3 years for evaluation of recurrence. The follow-up time for patients who did not survive was defined as the duration between the dates of surgery and mortality. The Institutional Review Board at Seoul St. Mary’s Hospital approved the handling of tissue samples and the patient data in the present study. Written informed consent was obtained from the patients and patient anonymity was preserved throughout this study.

The mean patient age was 62.8 years (standard deviation, 11.7) and 328 of the patients (61.4%) were male. The tumor location was the right colon in 150 (28.1%) patients, the left colon in 178 patients (33.3%) and the rectum in 206 patients (38.6%). The numbers of patients with disease stages I, II, III and IV were 78 (14.6%), 162 (30.3%), 206 (38.6%) and 88 (16.5%), respectively, according to the American Joint Committee on Cancer tumor node metastasis (TNM) system (14). The tumor grade was well/moderately differentiated in 494 (92.5%) patients and poorly differentiated in 40 (7.5%) patients, by the World Health Organization tumor classifcation system (15). R0 resection was administered to 478 (89.5%) patients, and 97 (18.2%) patients received adjuvant chemotherapy. The median follow-up time was 42 months (range, 2–80). All data regarding the follow-up studies were evaluated on the basis of information available as of October, 2010.

### Tissue microarray generation

Tissue microarrays were constructed from archival formalin-fixed, paraffin-embedded carcinoma samples obtained from primary CRC specimens using a manual tissue arrayer (Quick-Ray Manual Tissue Microarrayer; Unitma Co., Ltd.; Seoul, Korea). For each sample, two areas rich in viable tumor cells (>80%) in the invasive front and in the tumor center were identified by light microscopic examination of hematoxylin and eosin-stained sections, and were marked for use in the tissue microarrays. Tissue cylinders with a diameter of 2 mm were punched from the previously marked tumor area of each block (the donor block) and inserted into a recipient paraffin block, resulting in a 6×10 array.

### Immunohistochemical staining

Immunohistochemistry for CB1 was performed on paraffin-embedded tissue sections of the tissue microarrays. Immunohistochemical reactions were conducted using a Polink-2 HRP Plus Broad detection system (Golden Bridge International; Mukilteo, WA, USA) according to the manufacturer’s instructions. Briefly, the tissue sections were deparaffinized and quenched with 3% hydrogen peroxide in methanol for 10 min. Antigen retrieval was then conducted using 0.01 mol/l citrate buffer (pH 6.0) by heating the sample in a microwave pressure cooker for 20 min. The sections were incubated with rabbit polyclonal CB1 antibody (1:50; Cat. No. 23703; Abcam; Cambridge, UK) (10,13) at room temperature for 30 min, followed by incubation with Broad Antibody Enhancer for 10 min and then with Polymer-HRP for 10 min (Golden Bridge International, Inc., Mukilteo, WA, USA). The peroxidase reaction was developed using 3′3-diaminobenzidine tetrahydrochloride as the chromogen. Negative controls included substitution of the primary antibodies with normal rabbit IgG of the same concentration as the primary antibodies.

### Scoring of immunohistochemistry

Each preserved specimen was examined by a pathologist (C.K.J.) who was blind to the clinical status of the patients. The immunoreactive area for CB1 was scored as 0 (0%), 1 (<33%), 2 (33–66%) or 3 (>66%), as discussed previously by Michalski *et al*(11). Using a simple median split, i.e., <3 or =3, immunoreactivity of CB1 expression was categorized as low or high under light microscopy. When the pathologist had scored all the samples, these were repeatedly measured using the same procedure, but without accessing any previous data. Cases with different scores were then evaluated once more, also without knowledge of the previous results. The final scores were then entered into the database for analysis by another investigator (J.M.P.).

### Statistical analysis

Continuous data are presented as the mean ± standard deviation (SD), and categorical data are presented as quantities and proportions. To evaluate the difference between the groups of patients according to the immunoreactivity of CB1 receptor expression, the χ^2^ test was used for categorical data, and the two-sample independent t-test was used for continuous variables. Cumulative survival curves for patients according to CB1 immunoreactivity were determined by the Kaplan-Meier method, and differences between the groups were compared using the log-rank test. Univariate and multivariate analyses were performed with the Cox proportional hazard regression model to determine factors related to overall survival. All statistical analyses were performed using Statistical Analysis Software (SAS; SAS Institute; Cary, NC, USA). P<0.05 was considered to indicate a statistically significant difference.

## Results

### Immunohistochemical evaluation of CB1 expression

**Figure 1:**
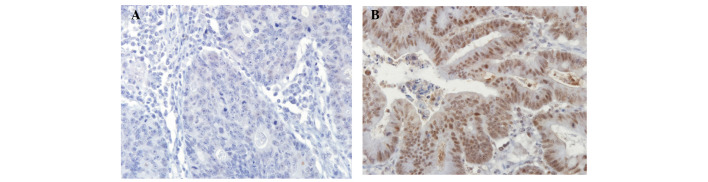
Examples of immunohistochemical staining of the cannabinoid type 1 (CB1) receptor in human colorectal cancer. Tumor cells exhibit low (A) and high (B) expression of the CB1 receptor. Original magnification, ×400.

**Figure 2:**
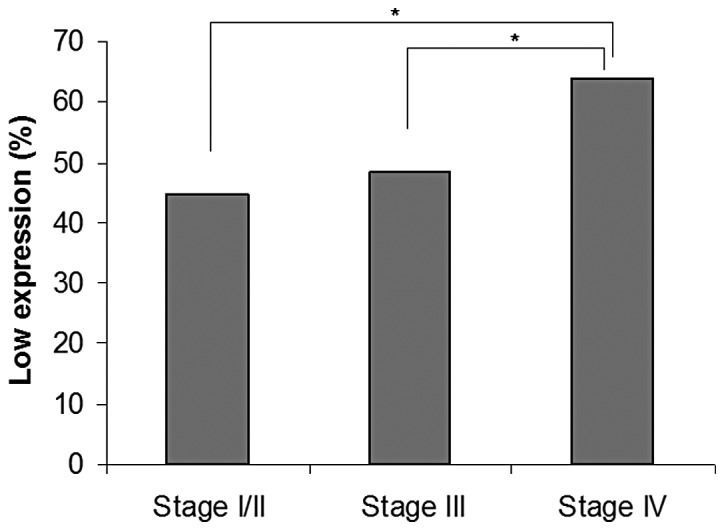
Cannabinoid type 1 (CB1) expression according to tumor stage. Compared to stage I/II or III, low CB1 expression is demonstrated more frequently in stage IV colorectal cancer. ^*^P<0.01.

Tumor cells with positive cytoplasmic staining for CB1 are illustrated in Fig. 1. Positive immunoreactivity for the CB1 receptor was observed in 409 patients (76.6%). The area of immunoreactive tumor cells was zero in 125 patients (23.4%), <33% in 24 patients (4.5%), 33–66% in 114 patients (21.3%) and >66% in 271 patients (50.7%). The proportion of low expression was significantly higher in stage IV than in stage I/II or III cancer (P<0.01 for both; Fig. 2). The expression level of the CB1 receptor at the invasive front was similar to that in the specimens from the tumor center (P=NS).

### Association of CB1 expression with clinicopathological findings

The correlations between CB1 receptor expression and the clinicopathological characteristics are summarized in Table I. No significant differences were observed in age, gender, tumor size, histological differentiation, primary tumor site, depth of invasion and lymph node metastasis between high and low CB1 receptor expression in the immunostaining results. However, distant metastasis was significantly higher in the patients with low CB1 receptor expression compared with those classified as having high expression (P= 0.003). Accordingly, the frequency was shown to be different in stage IV cancer (P=0.023).

As the frequency of distant metastasis was different between the group with high and low CB1 receptor expression, we compared the clinicopathological characteristics in stage IV cancer (Table II). No significant differences were observed in age, gender, histological differentiation, tumor site and follow-up interval between high and low CB1 receptor expression. Additionally, no significant difference was observed in the number of R0 resections performed in patients with high and low CB1 receptor expression; 18 (56.3%) and 29 (51.8%), respectively (P=0.686).

### Survival according to cancer stage

**Figure 3:**
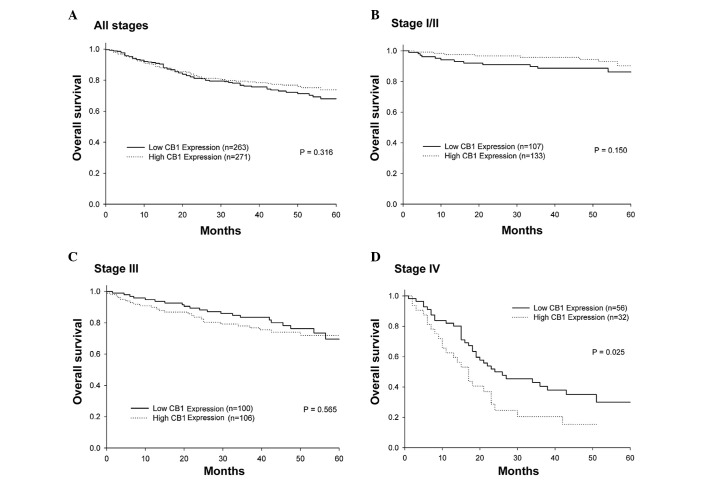
Influence of cannabinoid type 1 (CB1) receptor expression at surgery on overall survival by Kaplan-Meier plots. (A) All stages; (B) stage I/II; (C) stage III and (D) stage IV.

The Kaplan-Meier analysis of the data for a total of 534 patients revealed that the overall survival between the patients with high vs. low CB1 receptor expression in tumors was not significantly different (Fig. 3A) (log-rank test; P=0.316). In stage I/II and III cancers, the overall survival of patients with high vs. low CB1 receptor expression was not significantly different (Fig. 3B and C). The patients with high CB1 receptor expression in tumors had poorer outcomes than the patients with low expression (Fig. 3D). The hazard ratio (HR) was 1.782 with a 95% confidence interval (CI) of 1.049–2.988 (P=0.033).

### Factors affecting overall survival

The multivariate analysis of factors related to overall survival in stage IV is shown in Table III. A Cox proportional hazards regression analysis indicated that a high CB1 expression level, along with incomplete resection of the tumor and undifferentiated pathology was an independent variable associated with a poorer disease outcome in stage IV cancer (HR, 1.805; 95% CI, 1.042–3.094; P= 0.035).

## Discussion

The function of the CB1 receptor underlying the pathophysiology and outcome of CRCs has not been clearly delineated. In this study, we demonstrated that CB1 receptor expression is correlated with distant metastasis, but not with tumor invasion and lymph node metastasis, in CRC. In terms of the patient outcome, high CB1 receptor expression is correlated with poor survival in stage IV CRC, and is an independent prognostic factor even after adjusting for covariates. However, high CB1 receptor expression was not associated with the clinical outcomes in stage I/II and III CRC.

Cannabinoid molecules have been of significance in the study of potential cancer therapies, as they have demonstrated potential antitumor effects in cultured cell lines and in animal models. Previous studies have indicated that the majority of the effects of exogenous cannabinoids act through the activation of the CB1 receptor (6). In an endogenous system, downregulation of CB1 receptor expression was observed in neoplastic epithelial cells from colon cancer biopsies (16). The mechanism of this finding is explained by epigenetic silencing of the CB1-encoding gene that contributes to a loss of its trans cription (3), as is frequently found in the inactivation of tumor suppressor genes (17). However, the functions of the CB1 receptor gene and the mechanisms underlying the transcriptional regulation of the CB1 receptor are not clearly delineated. The present data revealed that CB1 receptor expression is downregulated as CRC progresses to a highly advanced stage, which is concordant with our hypothesis. This finding is also supported by a previous observation by Gustafsson *et al* that 64% of patients with stage IV CRC exhibited low immunoreactivity of the CB1 receptor (13).

Our data demonstrated that high CB1 receptor expression in CRC confers a poor prognosis for the patient. We had predicted that high CB1 receptor expression would lead to a better outcome in patients with CRC; however, the opposite result was observed. Notably, our observation was similar to that of the study by Gustafsson *et al,* in which CRC with a high intensity of the CB1 receptor was correlated with a shorter survival time than those with a low CB1 receptor intensity (13). It is difficult to clearly explain this observation. In general, the prognosis of CRC has been known to be correlated with the type, density and location of immune cells within the tumor (18), and certain studies have indicated that the presence of tumor-infiltrating lymphocytes within cancer cell nests and the tumor stroma is related to improved survival (19,20). CB1 has a pivotal role in modulating the immune response. A previous study demonstrated that mice in which the CB1-encoding gene had been knocked out exhibited a stronger inflammatory response in the colon compared with wild-type mice in response to treatment with pro-inflammatory agents (21), suggesting an immunomodulatory role of the CB1 receptor. Therefore, it may be speculated that tumors with low CB1 expression in the tumor tissue may elicit a low inflammatory environment. The study by Gustafsson *et al* revealed that CRC with low CB1 receptor intensity in the tumor front presented a higher number of infiltrating lymphocytes than CRC with high CB1 receptor intensity, with marginal statistical significance (13). The other possible explanation for this observation is that a high level of CB1 receptor expression is able to activate the pro-survival cellular pathway. CB1 receptors are coupled to a variety of signaling cascades, including cyclic AMP and activation of the extracellular signal-related kinase pathway (7), which are able to cause cell proliferation (22). Furthermore, a previous study indicated that activation of the CB1 receptor results in activation of the Akt signaling pathway and that cannabinoids only induced tumor cell apoptosis when this pathway was inhibited (23).

Another potential explanation is that CB1 receptor expression is a compensatory response for the endogenous cannabinoid level. This implies that a higher degree of endogenous cannabinoid in tumors favors apoptosis in cancer cells; this results in better survival and leads to downregulation of CB1 receptor expression in a compensatory way. Regarding this explanation, the level of endogenous cannabinoid-metabolizing enzymes, as well as the CB1 receptor, were analyzed in a pancreatic cancer study; however, this study failed to indicate the correlation between enzyme function and CB1 receptor level and patient survival (11). Studies in CRC to further explain this observation are necessary.

Notably, high CB1 receptor expression in stage IV CRC is an independent prognostic factor, even following adjustment for R0 resection, tumor differentiation and chemotherapy. A recent study indicated that the CB1 receptor antagonist rimonabant was able to control tumor growth (24). Therefore, it would be valuable to know whether patients with high CB1 receptor expression can be treated effectively with this receptor-blocking agent.

To our knowledge, four studies have investigated the correlation between CB1 receptor expression and disease outcome in cancer. Our data was supported by three of these studies, which demonstrated that high CB1 receptor immunoreactivity was correlated with a shorter survival time than low immunoreactivity in pancreatic, prostate and colorectal cancers (10,11,13). In contrast, disease-free survival in hepatocellular carcinoma was observed to be lower in patients with low CB1 receptor immunoreactivity than in those with high immunoreactivity (12). These discrepant results may indicate different roles of CB1 receptors that are dependent on the type of cancer.

The observation time and number of mortalities in our study may not have been sufficient to discern the survival difference according to the CB1 receptor expression level in stage I/II or III CRC; therefore, these factors may have caused different results from a previous study (13). However, we propose that the results of stage IV cancer portray the true role of CB1, as fatalities occurred during a relatively short time period. Furthermore, the multivariate analysis supports the important and independent association of the CB1 receptor with patient survival. In a previous study of patients with pancreatic cancer, which has a poorer prognosis than CRC, a difference in survival was observed according to the expression level of CB1 (11). Limitations of the present study include the status of microsatellite instability, the level of endogenous cannabinoids and the fact that the metabolizing enzymes of endogenous cannabinoids in tumors were not evaluated. The use of a large sample size of well-characterized patients with a long follow-up period may allow for the correlation between CB1 expression and the disease outcomes to be determined.

In conclusion, the present study demonstrated that high CB1 receptor expression is independently correlated with decreased survival in stage IV CRC. Future studies investigating other components of the endogenous cannabinoid system are required to clarify the exact mechanism and the correlation with endogenous cannabinoids.

## Figures and Tables

**Table I:** Characteristics of the tumor tissue samples with high and low expression of the cannabinoid type I (CB1) receptor.

	Expression of the CB1 receptor		
Categories	Low (n=263) (%)	High (n=271) (%)	P-value
Mean age ± SD (years)	62.3±11.7	63.3±11.7	0.319
Gender			
Male	163 (62.0)	165 (60.9)	0.796
Female	100 (38.0)	106 (39.1)	
Tumor size	5.0±1.8	5.4±2.4	0.168
Histological differentiation^a^			
Differentiated	245 (93.2)	249 (91.9)	0.576
Undifferentiated	18 (6.8)	22 (8.1)	
Tumor site			
Right	71 (27.0)	79 (29.2)	0.157
Left	80 (30.4)	98 (36.2)	
Rectum	112 (42.6)	94 (34.7)	
Depth of invasion			0.888
T1	7 (2.7)	8 (3.0)	
T2	32 (12.2)	39 (14.4)	
T3	167 (63.5)	167 (61.6)	
T4	57 (21.7)	57 (21.0)	
Lymph node metastasis			
N0	119 (45.2)	137 (50.6)	0.112
N1	67 (25.5)	76 (28.0)	
N2	77 (29.3)	58 (21.4)	
Distant metastasis			
M0	207 (78.7)	239 (88.2)	0.003
M1	56 (21.3)	32 (11.8)	
TNM stage			0.023
I	36 (13.7)	42 (15.5)	
II	71 (27.0)	91 (33.6)	
III	100 (38.0)	106 (39.1)	
IV	56 (21.3)	32 (11.8)	

a In cases of mixed histological differentiation, the more poorly differentiated tumor was selected. TNM, tumor node metastasis.

**Table II:** Clinicopathological characteristics of patients with stage IV colorectal cancer in relation to expression of cannabinoid type I (CB1) receptor immunoreactivity.

	Expression of the CB1 receptor		
Categories	Low (n=56) (%)	High (n=32) (%)	P-value
Mean age ± SD (years)	59.1±11.9	61.5±9.3	0.293
Gender			
Male	34 (60.7)	20 (62.5)	0.869
Female	22 (39.3)	12 (37.5)	
Histological differentiation^a^			0.210
Differentiated	48 (85.7)	24 (75.0)	
Undifferentiated	8 (14.3)	8 (25.0)	
Tumor site			0.187
Right	20 (35.7)	8 (25.0)	
Left	17 (30.4)	16 (50.0)	
Rectum	19 (33.9)	8 (25.0)	
Adjuvant chemotherapy	18 (32.1)	7 (21.9)	0.304
R0 resection	29 (51.8)	18 (56.3)	0.686
Follow-up (months)^b^	22.5 (15–40.3)	17 (8.3–32.3)	0.134

a In cases of mixed differentiation, the more poorly differentiated tumor was selected.

b Median and interquartile range, compared by Kruskal-Wallis test.

**Table III:** Multivariate analysis of tumor variables and overall survival in stage IV cancer (n=88).

Variable	HR (95% CI)	P-value
Age	1.000 (0.974–1.021)	0.7803
Expression of CB1 receptor (≥66 vs. <66%)	1.805 (1.042–3.094)	0.0353
Resection (R1/R2 vs. R0)	4.506 (1.933–13.152)	0.0002
Chemotherapy (no vs. yes)	2.000 (0.761–6.213)	0.1653
Differentiation (undifferentiated vs. differentiated)	2.755 (1.449–4.987)	0.0027

CB1, cannabinoid type 1; HR, hazard ratio; CI, confidence interval.
